# Crystal structures of 3-halo-2-organochalcogenylbenzo[*b*]chalcogenophenes

**DOI:** 10.1107/S2056989022000962

**Published:** 2022-02-03

**Authors:** Eduardo Q. Luz, Francielli S. Santana, Gabriel L. Silverio, Suelen C. M. C. Tullio, Bianca Iodice, Liziê D. T. Prola, Ronilson V. Barbosa, Daniel S. Rampon

**Affiliations:** a Laboratory of Polymers and Catalysis (LaPoCa), Department of Chemistry, Federal University of Paraná-UFPR, PO Box 19061, Curitiba, PR, 81531-980, Brazil; b Department of Chemistry, Federal University of Paraná-UFPR, PO Box 19061, Curitiba, PR, 81531-980, Brazil; c Department of Biology, East Carolina University, Greenville, North Carolina, USA; d IOTO USA – 1997N Greene Street – Greenville, NC 27834, USA; e Department of Chemistry and Biology, Federal University of Technology - Paraná, Rua Deputado Heitor de Alencar Furtado, 5000, 81280-340, Curitiba, Brazil; f IOTO INTERNATIONAL - Rodovia Gumercindo Boza 20088 – Campo Magro – PR, 83535-000, Brazil

**Keywords:** crystal structure, benzo[*b*]chalcogenophenes, intra­molecular orbital inter­action

## Abstract

The structure of the title compounds 3-bromo-2-(phenyl­sulfan­yl)benzo[*b*]thiophene (C_14_H_9_BrS_2_; **1**), 3-iodo-2-(phenyl­sulfan­yl)benzo[*b*]thio­phene (C_14_H_9_IS_2_; **2**), 3-bromo-2-(phenyl­selan­yl)benzo[*b*]seleno­phene (C_14_H_9_BrSe_2_; **3**), and 3-iodo-2-(phenyl­selan­yl)benzo[*b*]seleno­phene (C_14_H_9_ISe_2_; **4**) were determined by single-crystal X-ray diffraction; all structures presented monoclinic (*P*2_1_/*c*) symmetry. The phenyl group is distant from the halogen atom to minimize the steric hindrance repulsion for all structures. Moreover, the structures of **3** and **4** show an almost linear alignment of halogen–selenium–carbon atoms arising from the intra­molecular orbital inter­action between a lone pair of electrons on the halogen atom and the anti­bonding σ*_Se–C_ orbital (*n*_halogen_→σ*_Se–C_). This inter­action leads to significant differences in the three-dimensional packing of the mol­ecules, which are assembled through π–π and C—H⋯π inter­actions. These data provide a better comprehension of the inter­molecular packing in benzo[*b*]chalcogenophenes, which is relevant for optoelectronic applications.

## Abstract 2

The structure of four benzo[*b*]chalcogenophenes are described. The presence of a phenyl­selanyl group at a vicinal position of bromide or iodine triggers a stabilizing intra­molecular orbital inter­action between a lone pair of electrons of a halogen atom and the anti­bonding σ*_(Se–C)_ orbital (*n*_halogen_–σ*_(Se–C_), resulting in the almost linear alignment of the halogen–selenium–carbon atoms that changes the conformation and also the three-dimensional packing.

## Chemical context

Chalcogenophenes derivatives are an attractive synthetic class of compounds with a wide range of relevant applications in medicinal chemistry (Keri *et al.*, 2017 ▸; Mahmoud *et al.*, 2017 ▸; Paegle *et al.*, 2016 ▸), electrochemistry (Wei *et al.*, 2017 ▸; Shahjad *et al.*, 2017 ▸), agrochemistry (Zani *et al.*, 2004 ▸) and as organic semiconductors (Yang *et al.*, 2018 ▸; Ostroverkhova, 2016 ▸). π-extended benzo[*b*]chalcogenophenes derivatives have been widely studied as improved materials for optoelectronic devices such as organic photovoltaic cells (OPVs) (Ashraf *et al.*, 2015 ▸; An *et al.*, 2018 ▸; Chen *et al.*, 2017 ▸), liquid-crystal displays (LCD) (Ghosh & Lehmann, 2017 ▸; Mei *et al.*, 2013 ▸), organic light-emitting diodes (OLEDs) (Grimsdale *et al.*, 2009 ▸; Zampetti *et al.*, 2017 ▸; Arsenyan *et al.*, 2019 ▸), and in organic field-effect transistors (OFETs) (Lee *et al.*, 2019 ▸; Tisovský *et al.*, 2019 ▸). Benzo[*b*]chalcogenophenes derivatives also show relevant biological activities as anti-tumor (Arsenyan *et al.*, 2011 ▸) and anti-inflammatory agents (Shah *et al.*, 2018 ▸). As part of our continuing work on benzo[*b*]chalcogenophenes (Luz *et al.*, 2021 ▸), we report herein the crystallographic structural comparison of four 3-halo-2-(organochalcogen­yl)benzo[*b*]chalcogenophene derivatives. 
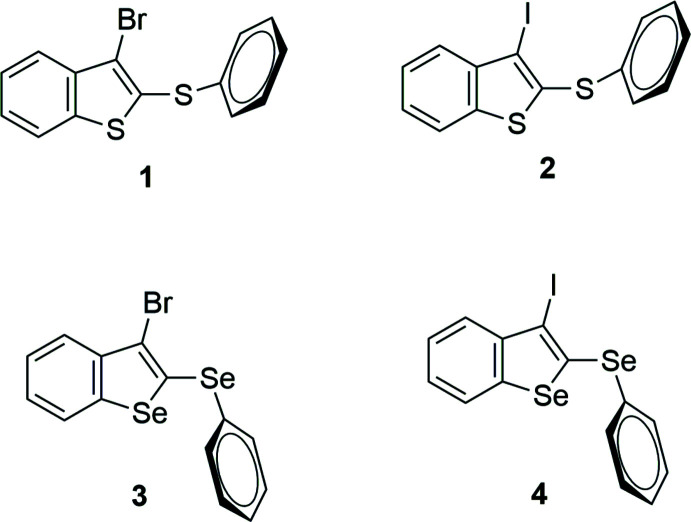


## Structural commentary

**Figure 1:**
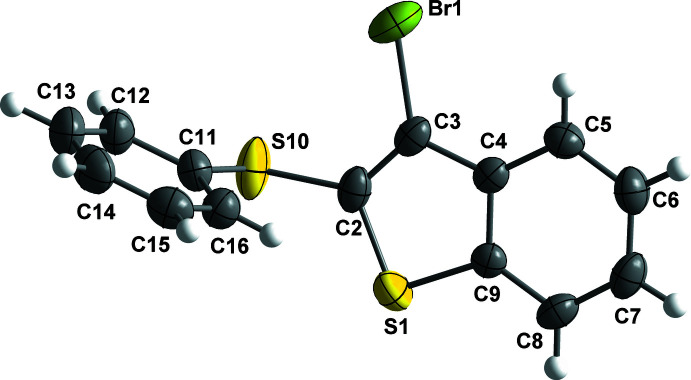
The mol­ecular structure of 3-bromo-2-(phenyl­sulfan­yl)benzo[*b*]thio­phene (**1**), with displacement ellipsoids drawn at the 50% probability level.

**Figure 3:**
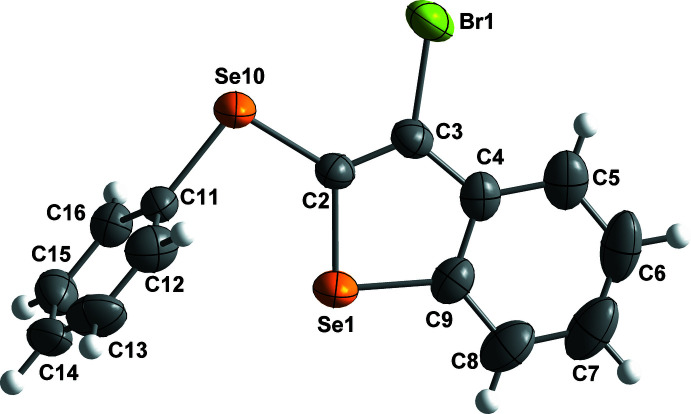
The mol­ecular structure of 3-bromo-2-(phenyl­selan­yl)benzo[*b*]seleno­phene (**3**), with displacement ellipsoids drawn at the 50% probability level.

The four organic compounds crystallize in the monoclinic *P*2_1_/*c* space group, and all atoms occupy unique positions. Compounds **1** and **2** are isostructural containing an identical 3-halo-2-(phenysulfan­yl)benzo[*b*]thio­phene unit with bro­mine (**1**) or iodine (**2**) at the C3 position of the benzo[*b*]thio­phene ring (Figs. 1 ▸ and 2 ▸). The isostructural compounds **3** and **4** also contain identical 3-halo-2-(phenyl­selan­yl)benzo[*b*]seleno­phene units with bromine (**3**) or iodine (**4**) at the C3 position of the benzo[*b*]seleno­phene ring (Figs. 3 ▸ and 4 ▸). The respective benzo[*b*]chalcogenophene rings and the phenyl­sulfanyl and phenyselanyl groups are planar. As expected, the carbon–selenium bonds in mol­ecules **3** and **4** are longer than the respective carbon–sulfur bonds in mol­ecules **1** and **2**.

**Figure 5:**
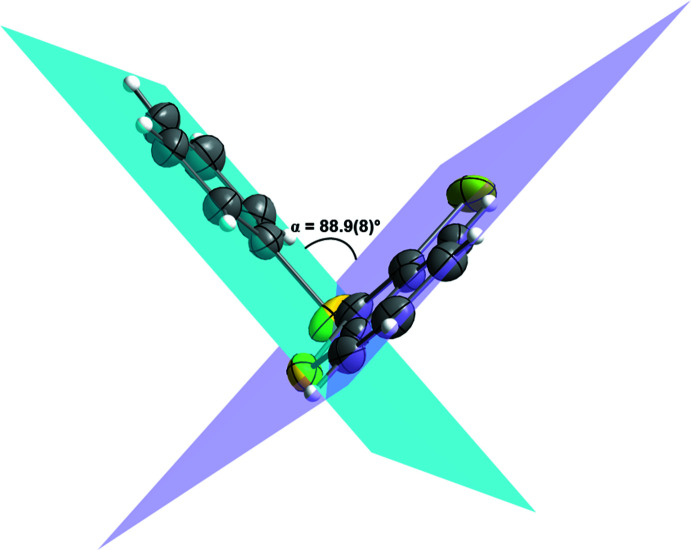
Representation of the inter­planar angle (α) between the planes containing the phenyl­sulfanyl, blue plane, and the benzo[*b*]thio­phene, purple plane, groups for 3-bromo-2-(phenyl­sulfan­yl)benzo[*b*]thio­phene (**1**). Displacement ellipsoids are drawn at the 50% probability level. Gray: carbon; yellow: sulfur; light green: bromine; white: hydrogen.

**Figure 7:**
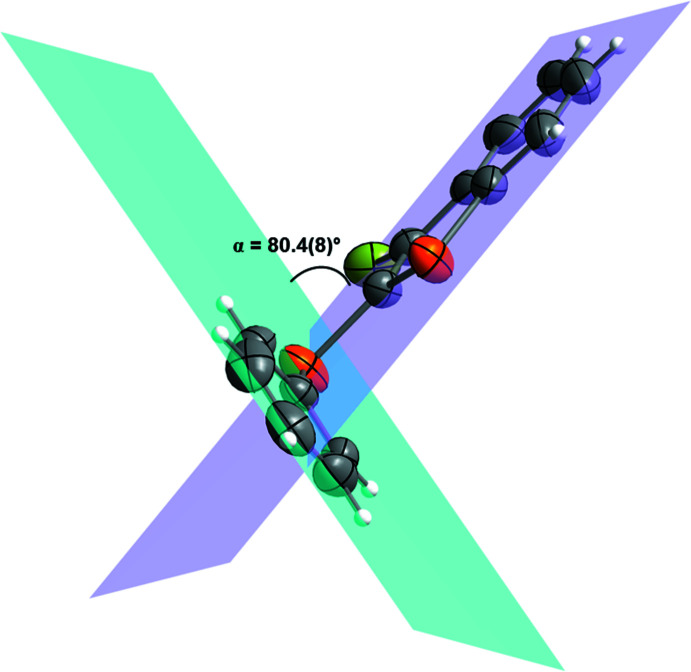
Representation of the inter­planar angle (α) between the planes containing the phenyl­selanyl, blue plane, and the benzo[*b*]seleno­phene, purple plane, groups for 3-bromo-2-(phenyl­selan­yl)benzo[*b*]seleno­phene (**3**). Displacement ellipsoids are drawn at the 50% probability level. Gray: carbon; orange: selenium; light green: bromine; white: hydrogen.

Conformational changes are observed when we compare mol­ecules **1** and **2** containing sulfur atoms with mol­ecules **3** and **4** containing selenium atoms, as described below. In mol­ecules **1** and **2**, the benzo[*b*]thio­phene ring is twisted away from the plane of phenyl­sulfanyl group showing inter­planar angles of 88.9 (8) and 87.9 (6)°, respectively (Figs. 5 ▸ and 6 ▸). Additionally, for **1** and **2** the S1—C2—S10—C11 torsion angles are −97.56 (14) and 98.17 (15)°, respectively. Mol­ecules **3** and **4** also show the benzo[*b*]seleno­phene ring twisted away from the plane of the phenyl­selanyl group with inter­planar angles of 80.4 (8) and 79.7 (7)°, respectively (Figs. 7 ▸ and 8 ▸). Conversely, the torsion angles (Se1—C2—Se10—C11) in mol­ecules **3** and **4** are 1.9 (3) and −4.0 (3)°, respectively, quite different than the S1—C2—S10—C11 torsion angles in mol­ecules **1** and **2**. It is clear that the coplanarity between the phenyl and benzo[*b*]chalcogenophene rings is avoided in both pairs of mol­ecules to minimize steric hindrance. This structural arrangement is reinforced by the presence of the halogen atom at the C3 position of the benzo[*b*]chalcogenophene ring (Figs. 1 ▸, 2 ▸, 3 ▸ and 4 ▸). Nevertheless, there is an almost linear alignment between the atoms Br1—Se10—C11 (**3**) and I1—Se10—C11 (**4**), which cannot be explained by steric factors alone. For instance, if we consider merely the higher steric hindrance between the phenyl and benzo[*b*]seleno­phene rings arising from the lower intrinsic C11—Se10–C2 angle directing the conformation of mol­ecules **3** and **4**, the almost linear alignment between the atoms Br1—Se10—C11 (**3**) and I1—Se10—C11 (**4**) is still not fully understood. We have observed that the inter­atomic distances between the chalcogen and the halogen atoms [S10⋯Br1 (**1**) = 3.5061 (8) Å, S10⋯I1 (**2**) = 3.6310 (7) Å, Se10⋯Br (**3**) = 3.4196 (7) Å, Se10⋯-I (**4**) = 3.5260 (7) Å] are 0.14, 0.15, 0.33 and 0.35 Å shorter than the sum of the van der Waals radii of the respective two atoms in mol­ecules **1**, **2**, **3**, and **4**, respectively (Bondi, 1964 ▸). The shorter inter­atomic distances Se10⋯Br and Se10⋯I and the remarkably almost linear alignment of the atoms in **3** [C11—Se10⋯Br1 = 152.95 (9)°] and in **4** [C11—Se10⋯I1 = 156.52 (1)°] when compared to mol­ecules **1** [C11—S10⋯Br1 = 93.01 (7)°] and **2** [C11—S10⋯I1 = 91.35 (7)°] indicate a stabilizing intra­molecular orbital inter­action (3-center-4-electrons, 3*c*–4*e*) between a lone pair of electrons of the halogen atom and the anti­bonding σ*_Se–C11_ orbital (*n*_halogen_→σ*_Se–C11_) (Mukherjee, 2010 ▸). The lower energy of the anti­bonding σ*_Se–C11_ orbital makes it a better acceptor when compared to the higher energy anti­bonding σ*_S–C11_ orbital, therefore making the intra­molecular *n*_halogen_→σ*_Se–C11_ orbital inter­action in mol­ecules **3** and **4** strong enough to change their mol­ecular conformation.

## Supra­molecular features

**Figure 9:**
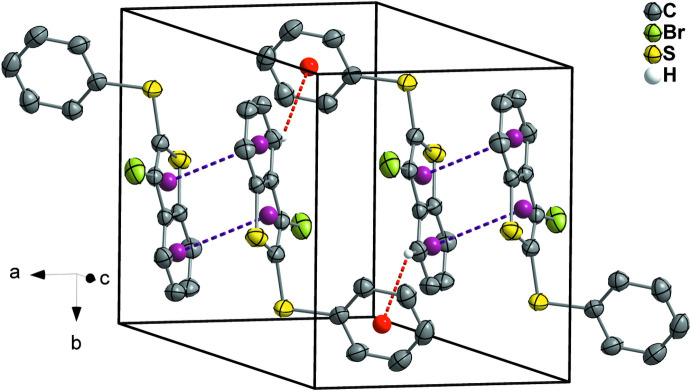
Representation of some mol­ecules of 3-bromo-2-(phenyl­sulfan­yl)benzo[*b*]seleno­phene (**1**) viewed approximately down the *c* axis of the unit cell. The red dashed lines represent C—H⋯π inter­actions involving the H5 atom of the benzo[*b*]thio­pehene ring with an adjacent phenyl­sulfanyl group; the purple dashed lines represent π–π stacking inter­actions between adjacent benzo[*b*]thio­pehene rings. Displacement ellipsoids are drawn at the 50% probability level. The hydrogen atoms, except for H4, are omitted for clarity. Red and purple spheres represent the centroids of the respective organic groups.

**Figure 10:**
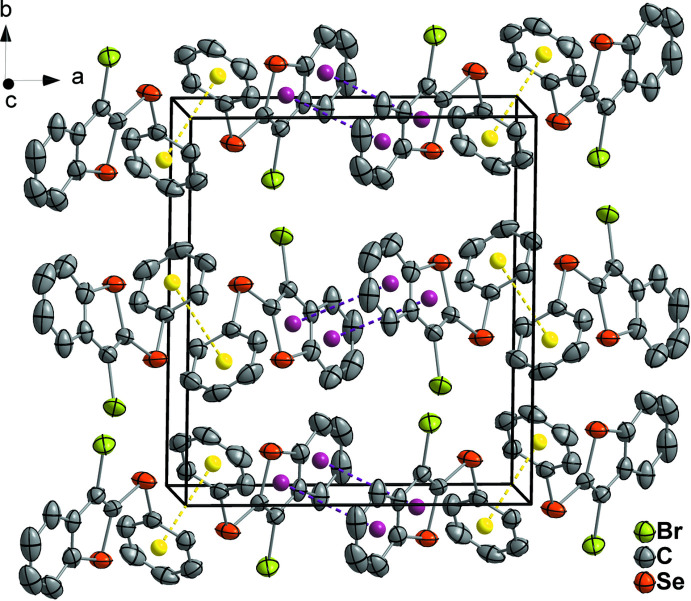
Representation of the mol­ecules of 3-bromo-2-(phenyl­selan­yl)benzo[*b*]seleno­phene (**3**) viewed down the *c* axis of the unit cell. The purple and yellow dashed lines represent π–π stacking inter­actions between adjacent benzo[*b*]thio­pehene rings and between adjacent phenyl­sulfanyl groups, respectively. Displacement ellipsoids are drawn at the 50% probability level. The hydrogen atoms were omitted to clarity. Purple and yellow spheres represent the centroids of the respective organic groups.

The crystals of organic compounds **1** and **2** are related by an inversion center and assembled through C—H⋯π inter­molecular inter­actions along the *b*-axis direction (Fig. 9 ▸). The weak C—H⋯π inter­actions are between the H5 atom and the centroid formed by atoms C11–C16 of the phenyl­sulfanyl group. The distances and angles comprising these contacts are 2.97 (2) Å, 137.1 (2)° for **1** and 2.93 (3) Å, 138.4 (2)° for **2**. The structures **1** and **2** also show π–π stacking inter­actions between adjacent benzo[*b*]thio­phene rings along the *c*-axis direction with centroid–centroid distances of 3.7166 (2) and 3.7602 (4) Å for **1** and **2**, respectively (Fig. 9 ▸, for **1**). On the other hand, in compounds **3** and **4** C—H⋯π inter­actions are not present. However, π–π stacking inter­actions involving adjacent benzo[*b*]thio­phene rings are present along the *a-*axis direction, with centroid–centroid distances of 3.8139 (3) Å and 3.8772 (1) Å, respectively. Furthermore, π–π stacking inter­actions are observed along the *b-*axis direction between phenyl­sulfanyl groups related by an inversion center, with centroid–centroid distances of 3.6644 (2) and 3.7351 (1) Å for **3** and **4**, respectively (Fig. 10 ▸, for **3**).

## Database survey

Several crystal structures of benzo[*b*]chalcogenophenes derivatives have been published. To the best of our knowledge, there are no studies about chalcogen atoms attached directly at position 2 of the benzo[*b*]chalcogenophene ring. With regard to benzo[*b*]thio­phenes, Xu *et al.* (2017 ▸) described the structure of 3-(aryl­sulfon­yl)benzo[*b*]thio­phene obtained by single-crystal X-ray diffraction. Additionally, Ramesh *et al.* (2016 ▸) reported the structures of 6-fluoro-2,2-(diphen­yl)benzo[*b*]thio­phene and 6-isopropyl-2,2-(diphen­yl)benzo[*b*]thio­phene obtained by single-crystal X-ray diffraction studies.

## Synthesis and crystallization

The structures reported here were obtained by the one-pot synthesis of 3-halo-2-organochalcogenylbenzo[*b*]chalcogenophenes from 1-(2,2-di­bromo­vin­yl)-2-organochalcogenyl­benz­enes. By this method, a series of 2,3-disubstituted benzo[*b*]chalcogenophenes were prepared in yields of *ca* 80% (Luz *et al.*, 2021 ▸). The title compounds were prepared as follows:

**3-Bromo-2-(phenyl­sulfan­yl)benzo[** ***b*** **]thio­phene (1)**

To a Schlenk tube containing 1-(2,2-di­bromo­vin­yl)-2-butyl­sulfanyl­benzene (0.25 mmol, 1.0 equiv.), diphenyl di­sulfide (0.125 mmol, 1.0 equiv.) was added in dry dimethyl sulfoxide (2.0 mL) followed by the addition of cesium carbon­ate (0.244 g, 0.75 mmol, 3.0 equiv.). The reaction system was heated to 383 K and stirred for 1.5 h. Then, the reaction mixture was cooled to room temperature and 2.5 equivalents of NBS (*N*-bromo­succinimide) in 2 mL of di­chloro­methane were slowly added (2.0 min) into the system. The reaction mixture was stirred at room temperature for 2 h. After this, the reaction solution was diluted in saturated thio­sulfate solution (20 mL) and washed with ethyl acetate (3 × 10 mL). The organic phase was dried over magnesium sulfate and concentrated under reduced pressure. The product was further purified by flash chromatography using hexane as eluent. Colorless needle-shaped single crystals of **1** suitable for X-ray analysis were grown by slow evaporation of a concentrated ethyl acetate solution over several days at room temperature. Yield: 0.066 g (82%); withe solid, m.p. 337–340 K. ^1^H NMR (CDCl_3_, 400 MHz) *δ* (ppm) = 7.77–7.75 (*m*, 1 H); 7.70–7.68 (*m*, 1H); 7.58–7.55 (*m*, 2H); 7.44–7.40 (*m*, 1H); 7.36–7.30 (*m*, 4H). ^13^C{^1^H} NMR (CDCl_3_, 100 MHz) *δ* (ppm) = 141.1, 138.5, 135.9, 133.1, 129.5, 128.3, 126.4, 125.4, 125.2, 123.3, 121.9, 114.4. MS (Rel. Int.) *m*/*z*: 321 (84.0), 241 (100), 210 (63.4), 77 (54.8) HRMS: Calculated mass for C_14_H_10_BrS_2_ [*M*]^+^: 321.9302, found: 321.9310.

**3-Iodo-2-(phenyl­sulfan­yl)benzo[** ***b*** **]thio­phene (2)**

The first step for obtaining **2** was analogous to that described for **1**. The reaction mixture was cooled to room temperature and 1.5 equivalents of I_2_ in 2 mL of di­chloro­methane were slowly added (2.0 min) into the system. The reaction mixture was stirred at room temperature for 3.5 h. After this, the reaction solution was diluted in saturated sodium thio­sulfate solution (20 mL) and washed with ethyl acetate (3 × 10 mL). The organic phase was dried over magnesium sulfate and concentrated under reduced pressure. The product was further purified by flash chromatography using hexane as eluent. Colorless needle-shaped single crystals of **2** were obtained in the same way of **1**. Yield: 0.073 g (79%); yellow solid, m.p. 325–327K. ^1^H NMR (CDCl_3_, 400 MHz) *δ* (ppm) = 7.72 (*d*, *J* = 8.0 Hz, 1 H); 7.66 (*d*, *J* = 7.6 Hz, 1 H); 7.44–7.40 (*m*, 2H); 7.34–7.21 (*m*, 5H). ^13^C{^1^H} NMR (CDCl_3_, 100 MHz) *δ* (ppm) = 141.5, 141.2, 136.5, 134.7, 130.3, 129.3, 127.7, 126.2, 126.0, 125.5, 122.1, 90.3. MS (Rel. Int.) *m*/*z*: 368 (94.3), 240 (100), 120 (50.3), 77 (10.5). HRMS: Calculated mass for C_14_H_9_IS_2_ [*M*]^+^: 367.9185, found: 367.9188.

**3-Bromo-2-(phenyl­seln­yl)benzo[** ***b*** **]seleno­phene (3)**

To a Schlenk tube containing 1-(2,2-di­bromo­vin­yl)-2-butyl­selanyl­benzene (0.25 mmol, 1.0 equiv.), diphenyl diselen­ide (0.125 mmol, 1.0 equiv.) was added in dry dimethyl sulfoxide (2.0 mL) followed by the addition of cesium carbonate (0.244 g, 0.75 mmol, 3.0 equiv.). The reaction system was heated to 384 K and stirred for 0.5 h. Then, the reaction mixture was cooled to room temperature and 2.5 equivalents of NBS (*N*-bromo­succinimide) in 2 mL of di­chloro­methane were slowly added (2.0 min) into the system. The reaction mixture was stirred at room temperature for 1 h. After this, the reaction solution was diluted in saturated sodium thio­sulfate solution (20 mL) and washed with ethyl acetate (3 × 10 mL). The organic phase was dried over magnesium sulfate and concentrated under reduced pressure. The product were further purified by flash chromatography using hexane as eluent. Colorless needle-shaped single crystals of **3** were obtained in the same way as **1**. Yield: 0.081 g (79%); yellow oil. ^1^H NMR (CDCl_3_, 400 MHz) *δ* (ppm) = 7.76 (*dd*, *J* = 8.1 and 1.0 Hz, 1H); 7.69–7.66 (*m*, 3H); 7.41–7.32 (*m*, 4H); 7.24 (*ddd*, *J* = 8.2, 7.3 and 1.3 Hz, 1H). ^13^C{^1^H} NMR (CDCl_3_, 100 MHz) *δ* (ppm) = 141.1, 140.6, 134.4, 129.6, 129.5, 129.4, 129.0, 125.4, 125.1, 125.0, 124.9, 112.9. MS (Rel. Int.) *m*/*z*: 416 (96.8), 336 (100), 256 (42.4), 77 (62.0).

**3-Iodo-2-(phenyl­selan­yl)benzo[** ***b*** **]seleno­phene (4)**

The first step for obtaining **4** was similar to that described for **3**. The reaction mixture was cooled to room temperature and 1.5 equivalents of I_2_ in 2 mL of di­chloro­methane were slowly added (2.0 min) into the system. The reaction was stirred at room temperature for 1 h. After this, the reaction solution was diluted in saturated sodium thio­sulfate solution (20 mL) and washed with ethyl acetate (3 × 10 mL). The organic phase was dried over magnesium sulfate and concentrated under reduced pressure. The product was further purified by flash chromatography using hexane as eluent. Colorless needle single crystals of **4** were obtained in the same way of (**1**). Yield: 0.090 g (78%); Orange solid, m.p. 329–331 K. ^1^H NMR (CDCl_3_, 400 MHz) *δ* (ppm) = 7.73–7.64 (*m*, 4H); 7.41–7.33 (*m*, 4H); 7.23–7.20 (*m*, 1H). ^13^C{^1^H} NMR (CDCl_3_, 100 MHz) *δ* (ppm) = 143.9, 142.2, 134.9, 134.7, 129.8, 129.7, 129.1, 127.6, 125.7, 125.1, 88.9. MS (Rel. Int.) *m*/*z*: 464 (48.2), 334 (47.0), 256 (51.4), 77 (53.2), 51 (100).

## Refinement

Crystal data, data collection and structure refinement details are summarized in Table 1 ▸. Hydrogen atoms of **1**, **2** and **4** were located in difference-Fourier maps and were refined freely; the hydrogen atoms of **3** were included in idealized positions with aromatic C—H distances set to 0.93 Å and refined using a riding model *U*_iso_(H) = 1.2*U*_eq_(C).

## Supplementary Material

Crystal structure: contains datablock(s) 1, 2, 3, 4, shelx. DOI: 10.1107/S2056989022000962/jy2015sup1.cif

Structure factors: contains datablock(s) 1. DOI: 10.1107/S2056989022000962/jy20151sup2.hkl

Supporting information file. DOI: 10.1107/S2056989022000962/jy20151sup6.cml

Structure factors: contains datablock(s) 2. DOI: 10.1107/S2056989022000962/jy20152sup3.hkl

Supporting information file. DOI: 10.1107/S2056989022000962/jy20152sup7.cml

Structure factors: contains datablock(s) 3. DOI: 10.1107/S2056989022000962/jy20153sup4.hkl

Supporting information file. DOI: 10.1107/S2056989022000962/jy20153sup8.cml

Structure factors: contains datablock(s) 4. DOI: 10.1107/S2056989022000962/jy20154sup5.hkl

Supporting information file. DOI: 10.1107/S2056989022000962/jy20154sup9.cml

CCDC references: 2145011, 2145012, 2145013, 2145014

Additional supporting information:  crystallographic information; 3D view; checkCIF report

## Figures and Tables

**Figure 2:**
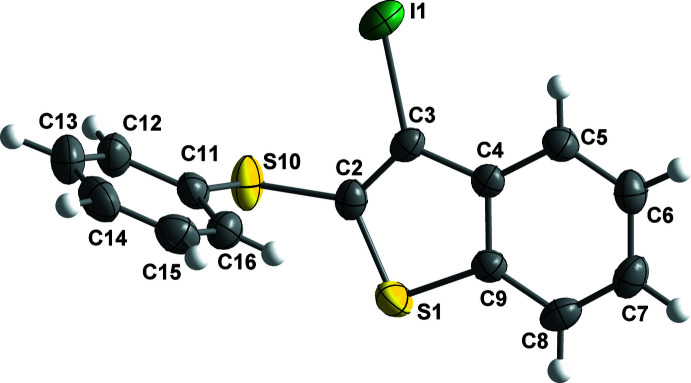
The mol­ecular structure of 3-iodo-2-(phenyl­sulfan­yl)benzo[*b*]thio­phene (**2**), with displacement ellipsoids drawn at the 50% probability level.

**Figure 4:**
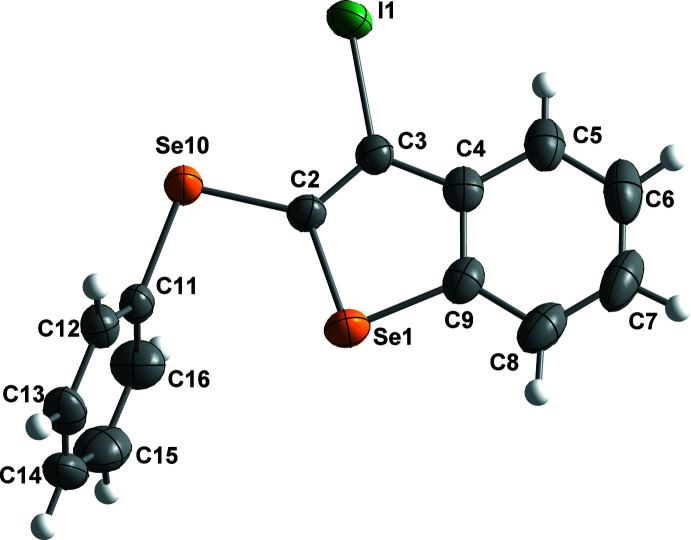
The mol­ecular structure of 3-iodo-2-(phenyl­selan­yl)benzo[*b*]seleno­phene (**4**), with displacement ellipsoids drawn at the 50% probability level.

**Figure 6:**
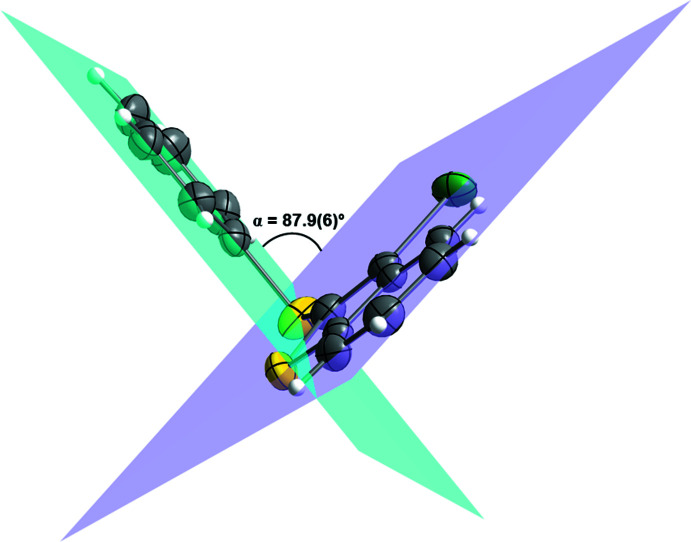
Representation of the inter­planar angle (α) between the planes containing the phenyl­sulfanyl, blue plane, and the benzo[*b*]thio­phene, purple plane, groups for 3-iodo-2-(phenyl­sulfan­yl)benzo[*b*]thio­phene (**2**). Displacement ellipsoids are drawn at the 50% probability level. Gray: carbon; yellow: sulfur; bluish green: iodine; white: hydrogen.

**Figure 8:**
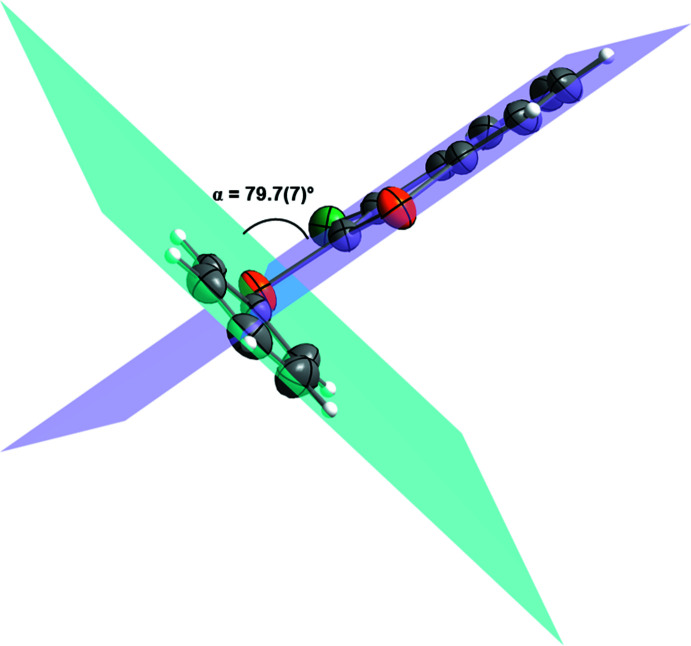
Representation of the inter­planar angle (α) between the planes containing the phenyl­selanyl, blue plane, and the benzo[*b*]seleno­phene, purple plane, groups for 3-iodo-2-(phenyl­selan­yl)benzo[*b*]seleno­phene (**4**). Displacement ellipsoids are drawn at the 50% probability level. Gray: carbon; orange: selenium; bluish green: iodine; white: hydrogen.

**Table 1:** Experimental details

	**1**	**2**	**3**	**4**
Crystal data				
Chemical formula	C_14_H_9_BrS_2_	C_14_H_9_IS_2_	C_14_H_9_BrSe_2_	C_14_H_9_ISe_2_
*M*_r_	321.24	368.23	415.04	462.03
Crystal system, space group	Monoclinic, *P*2_1_/*c*	Monoclinic, *P*2_1_/*c*	Monoclinic, *P*2_1_/*c*	Monoclinic, *P*2_1_/*c*
Temperature (K)	296	294	297	292
*a*, *b*, *c* (Å)	8.2471 (8), 9.9562 (8), 15.7601 (14)	8.4872 (3), 9.9629 (4), 15.6485 (7)	12.3864 (11), 13.6816 (11), 8.0982 (6)	12.9606 (6), 13.5999 (7), 8.0448 (4)
β (°)	98.967 (3)	97.052 (1)	96.398 (3)	95.585 (2)
*V* (Å^3^)	1278.2 (2)	1313.18 (9)	1363.82 (19)	1411.27 (12)
*Z*	4	4	4	4
Radiation type	Mo *K*α	Mo *K*α	Mo *K*α	Mo *K*α
μ (mm^−1^)	3.51	2.73	8.33	7.40
Crystal size (mm)	0.28 × 0.21 × 0.14	0.16 × 0.13 × 0.05	0.17 × 0.17 × 0.12	0.51 × 0.47 × 0.24

Data collection				
Diffractometer	Bruker D8 Venture/Photon 100 CMOS	Bruker D8 Venture/Photon 100 CMOS	Bruker D8 Venture/Photon 100 CMOS	Bruker D8 Venture/Photon 100 CMOS
Absorption correction	Multi-scan (*SADABS*; Krause *et al.*, 2015 ▸)	Multi-scan (*SADABS*; Krause *et al.*, 2015 ▸)	Multi-scan (*SADABS*; Krause *et al.*, 2015 ▸)	Multi-scan (*SADABS*; Krause *et al.*, 2015 ▸)
*T*_min_, *T*_max_	0.628, 0.746	0.690, 0.746	0.543, 0.746	0.390, 0.746
No. of measured, independent and observed [*I* > 2σ(*I*)] reflections	55020, 3078, 2559	56046, 3001, 2585	43875, 2976, 2208	54241, 3391, 2702
*R*_int_	0.041	0.045	0.060	0.054
(sin θ/λ)_max_ (Å^−1^)	0.660	0.650	0.639	0.660

Refinement				
*R*[*F* ^2^ > 2σ(*F* ^2^)], *wR*(*F* ^2^), *S*	0.029, 0.073, 1.04	0.022, 0.051, 1.08	0.035, 0.069, 1.03	0.037, 0.083, 1.04
No. of reflections	3078	3001	2976	3391
No. of parameters	190	190	154	190
H-atom treatment	All H-atom parameters refined	All H-atom parameters refined	H-atom parameters constrained	All H-atom parameters refined
Δρ_max_, Δρ_min_ (e Å^−3^)	0.41, −0.76	0.45, −0.86	0.93, −0.90	1.14, −1.82

Computer programs: *APEX3* (Bruker, 2015 ▸), *SAINT* (Bruker, 2002 ▸), *SHELXL97* (Sheldrick, 2008 ▸), *SHELXT* (Sheldrick, 2015*a* ▸), *SHELXL2018/3* (Sheldrick, 2015*b* ▸), *DIAMOND* (Brandenburg & Putz, 2006 ▸) and *WinGX* (Farrugia, 2012 ▸).

## supplementary crystallographic information

### 3-Bromo-2-(phenylsulfanyl)benzo[b]thiophene (1) Crystal data

**Table:**

C_14_H_9_BrS_2_	*F*(000) = 640
*M**_r_* = 321.24	*D*_x_ = 1.669 Mg m^−^^3^
Monoclinic, *P*2_1_/*c*	Mo *K*α radiation, λ = 0.71073 Å
*a* = 8.2471 (8) Å	Cell parameters from 9946 reflections
*b* = 9.9562 (8) Å	θ = 2.6–28.0°
*c* = 15.7601 (14) Å	µ = 3.51 mm^−^^1^
β = 98.967 (3)°	*T* = 296 K
*V* = 1278.2 (2) Å^3^	Parallelepiped, colourless
*Z* = 4	0.28 × 0.21 × 0.14 mm

### 3-Bromo-2-(phenylsulfanyl)benzo[b]thiophene (1) Data collection

**Table:**

Bruker D8 Venture/Photon 100 CMOS diffractometer	3078 independent reflections
Radiation source: fine-focus sealed tube	2559 reflections with *I* > 2σ(*I*)
Graphite monochromator	*R*_int_ = 0.041
Detector resolution: 10.4167 pixels mm^-1^	θ_max_ = 28.0°, θ_min_ = 3.2°
φ and ω scans	*h* = −10→10
Absorption correction: multi-scan (SADABS; Krause *et al.*, 2015)	*k* = −13→13
*T*_min_ = 0.628, *T*_max_ = 0.746	*l* = −20→20
55020 measured reflections	

### 3-Bromo-2-(phenylsulfanyl)benzo[b]thiophene (1) Refinement

**Table:**

Refinement on *F*^2^	Primary atom site location: dual
Least-squares matrix: full	Secondary atom site location: difference Fourier map
*R*[*F*^2^ > 2σ(*F*^2^)] = 0.029	Hydrogen site location: difference Fourier map
*wR*(*F*^2^) = 0.073	All H-atom parameters refined
*S* = 1.04	*w* = 1/[σ^2^(*F*_o_^2^) + (0.0347*P*)^2^ + 0.7059*P*] where *P* = (*F*_o_^2^ + 2*F*_c_^2^)/3
3078 reflections	(Δ/σ)_max_ < 0.001
190 parameters	Δρ_max_ = 0.41 e Å^−^^3^
0 restraints	Δρ_min_ = −0.76 e Å^−^^3^

### 3-Bromo-2-(phenylsulfanyl)benzo[b]thiophene (1) Special details

**Table:**

Geometry. All esds (except the esd in the dihedral angle between two l.s. planes) are estimated using the full covariance matrix. The cell esds are taken into account individually in the estimation of esds in distances, angles and torsion angles; correlations between esds in cell parameters are only used when they are defined by crystal symmetry. An approximate (isotropic) treatment of cell esds is used for estimating esds involving l.s. planes.

### 3-Bromo-2-(phenylsulfanyl)benzo[b]thiophene (1) Fractional atomic coordinates and isotropic or equivalent isotropic displacement parameters (Å^2^)

**Table:**

	*x*	*y*	*z*	*U*_iso_*/*U*_eq_	
C2	0.1651 (3)	0.3373 (2)	0.41693 (14)	0.0405 (5)	
C3	0.2308 (2)	0.4295 (2)	0.47529 (13)	0.0361 (4)	
C4	0.1731 (2)	0.56405 (19)	0.45645 (12)	0.0315 (4)	
C5	0.2140 (3)	0.6832 (2)	0.50152 (14)	0.0399 (4)	
C6	0.1421 (3)	0.8012 (2)	0.47034 (16)	0.0460 (5)	
C7	0.0310 (3)	0.8041 (2)	0.39437 (16)	0.0468 (5)	
C8	−0.0120 (3)	0.6888 (2)	0.34898 (14)	0.0426 (5)	
C9	0.0599 (2)	0.56820 (19)	0.38079 (12)	0.0325 (4)	
C11	0.3783 (3)	0.1481 (2)	0.36842 (14)	0.0392 (4)	
C12	0.4614 (3)	0.0261 (2)	0.38205 (16)	0.0487 (5)	
C13	0.6017 (3)	0.0046 (3)	0.34629 (17)	0.0562 (6)	
C14	0.6591 (3)	0.1024 (3)	0.29691 (18)	0.0578 (6)	
C15	0.5762 (3)	0.2225 (3)	0.28289 (16)	0.0512 (6)	
C16	0.4356 (3)	0.2460 (2)	0.31852 (14)	0.0435 (5)	
Br1	0.38366 (3)	0.38960 (3)	0.57285 (2)	0.05735 (10)	
S1	0.02548 (7)	0.41026 (6)	0.33534 (4)	0.04409 (13)	
S10	0.19752 (8)	0.16429 (6)	0.41634 (5)	0.05842 (19)	
H5	0.289 (3)	0.680 (2)	0.5496 (15)	0.042 (6)*	
H6	0.172 (3)	0.880 (3)	0.4980 (17)	0.053 (7)*	
H7	−0.009 (4)	0.887 (3)	0.3766 (18)	0.060 (8)*	
H8	−0.089 (3)	0.691 (3)	0.2997 (17)	0.055 (7)*	
H12	0.429 (3)	−0.039 (3)	0.4186 (17)	0.056 (7)*	
H13	0.660 (4)	−0.081 (3)	0.3556 (18)	0.065 (8)*	
H14	0.751 (4)	0.092 (3)	0.278 (2)	0.073 (9)*	
H15	0.613 (3)	0.286 (3)	0.2530 (18)	0.058 (8)*	
H16	0.379 (3)	0.325 (3)	0.3084 (17)	0.058 (8)*	

### 3-Bromo-2-(phenylsulfanyl)benzo[b]thiophene (1) Atomic displacement parameters (Å^2^)

**Table:**

	*U*^11^	*U*^22^	*U*^33^	*U*^12^	*U*^13^	*U*^23^
C2	0.0426 (11)	0.0318 (9)	0.0515 (12)	0.0038 (8)	0.0207 (9)	0.0049 (9)
C3	0.0300 (9)	0.0371 (10)	0.0432 (10)	0.0039 (7)	0.0116 (8)	0.0087 (8)
C4	0.0281 (8)	0.0343 (9)	0.0337 (9)	0.0004 (7)	0.0100 (7)	0.0047 (7)
C5	0.0368 (10)	0.0437 (11)	0.0389 (11)	−0.0035 (8)	0.0049 (8)	−0.0012 (9)
C6	0.0514 (13)	0.0329 (10)	0.0562 (13)	−0.0040 (9)	0.0160 (10)	−0.0046 (10)
C7	0.0511 (13)	0.0332 (10)	0.0583 (14)	0.0064 (9)	0.0157 (10)	0.0122 (10)
C8	0.0433 (11)	0.0431 (11)	0.0405 (11)	0.0038 (9)	0.0038 (9)	0.0130 (9)
C9	0.0339 (9)	0.0331 (9)	0.0321 (9)	−0.0002 (7)	0.0097 (7)	0.0025 (7)
C11	0.0416 (11)	0.0342 (10)	0.0429 (11)	−0.0005 (8)	0.0103 (8)	−0.0054 (8)
C12	0.0587 (14)	0.0356 (11)	0.0536 (13)	0.0060 (10)	0.0147 (11)	−0.0019 (10)
C13	0.0590 (15)	0.0477 (13)	0.0632 (15)	0.0141 (12)	0.0134 (12)	−0.0077 (12)
C14	0.0489 (14)	0.0706 (17)	0.0573 (15)	0.0079 (12)	0.0186 (11)	−0.0125 (13)
C15	0.0492 (13)	0.0611 (15)	0.0452 (12)	−0.0055 (11)	0.0135 (10)	0.0010 (11)
C16	0.0439 (11)	0.0415 (11)	0.0455 (12)	0.0020 (9)	0.0083 (9)	0.0024 (9)
Br1	0.03881 (13)	0.06510 (17)	0.06559 (17)	0.00695 (10)	0.00017 (10)	0.02733 (12)
S1	0.0519 (3)	0.0402 (3)	0.0404 (3)	−0.0055 (2)	0.0080 (2)	−0.0061 (2)
S10	0.0637 (4)	0.0289 (3)	0.0928 (5)	0.0022 (2)	0.0440 (4)	0.0058 (3)

### 3-Bromo-2-(phenylsulfanyl)benzo[b]thiophene (1) Geometric parameters (Å, º)

**Table:**

C2—C3	1.352 (3)	C8—H8	0.92 (3)
C2—S10	1.744 (2)	C9—S1	1.733 (2)
C2—S1	1.745 (2)	C11—C16	1.381 (3)
C3—C4	1.437 (3)	C11—C12	1.395 (3)
C3—Br1	1.873 (2)	C11—S10	1.781 (2)
C4—C9	1.396 (3)	C12—C13	1.380 (3)
C4—C5	1.397 (3)	C12—H12	0.93 (3)
C5—C6	1.373 (3)	C13—C14	1.376 (4)
C5—H5	0.90 (2)	C13—H13	0.98 (3)
C6—C7	1.390 (4)	C14—C15	1.377 (4)
C6—H6	0.91 (3)	C14—H14	0.87 (3)
C7—C8	1.369 (3)	C15—C16	1.385 (3)
C7—H7	0.92 (3)	C15—H15	0.87 (3)
C8—C9	1.397 (3)	C16—H16	0.91 (3)
			
C3—C2—S10	128.98 (18)	C4—C9—S1	111.78 (14)
C3—C2—S1	111.54 (15)	C8—C9—S1	126.76 (16)
S10—C2—S1	119.44 (14)	C16—C11—C12	119.9 (2)
C2—C3—C4	114.06 (18)	C16—C11—S10	124.14 (17)
C2—C3—Br1	124.17 (16)	C12—C11—S10	115.92 (17)
C4—C3—Br1	121.76 (15)	C13—C12—C11	119.7 (2)
C9—C4—C5	119.07 (18)	C13—C12—H12	118.7 (16)
C9—C4—C3	111.10 (17)	C11—C12—H12	121.4 (17)
C5—C4—C3	129.82 (18)	C14—C13—C12	120.3 (2)
C6—C5—C4	119.2 (2)	C14—C13—H13	120.0 (17)
C6—C5—H5	122.1 (16)	C12—C13—H13	119.7 (17)
C4—C5—H5	118.6 (16)	C13—C14—C15	119.9 (2)
C5—C6—C7	121.1 (2)	C13—C14—H14	121 (2)
C5—C6—H6	119.9 (17)	C15—C14—H14	119 (2)
C7—C6—H6	118.9 (17)	C14—C15—C16	120.5 (2)
C8—C7—C6	121.0 (2)	C14—C15—H15	120.8 (18)
C8—C7—H7	123.0 (18)	C16—C15—H15	118.7 (18)
C6—C7—H7	116.0 (18)	C11—C16—C15	119.6 (2)
C7—C8—C9	118.2 (2)	C11—C16—H16	119.7 (18)
C7—C8—H8	120.6 (18)	C15—C16—H16	120.7 (18)
C9—C8—H8	121.2 (18)	C9—S1—C2	91.51 (10)
C4—C9—C8	121.46 (19)	C2—S10—C11	103.35 (10)
			
S10—C2—C3—C4	−178.52 (15)	C7—C8—C9—S1	179.94 (17)
S1—C2—C3—C4	−0.7 (2)	C16—C11—C12—C13	0.7 (4)
S10—C2—C3—Br1	1.5 (3)	S10—C11—C12—C13	179.2 (2)
S1—C2—C3—Br1	179.28 (10)	C11—C12—C13—C14	−0.4 (4)
C2—C3—C4—C9	0.2 (2)	C12—C13—C14—C15	−0.1 (4)
Br1—C3—C4—C9	−179.83 (13)	C13—C14—C15—C16	0.3 (4)
C2—C3—C4—C5	−179.69 (19)	C12—C11—C16—C15	−0.5 (3)
Br1—C3—C4—C5	0.3 (3)	S10—C11—C16—C15	−178.85 (18)
C9—C4—C5—C6	0.0 (3)	C14—C15—C16—C11	0.0 (4)
C3—C4—C5—C6	179.8 (2)	C4—C9—S1—C2	−0.72 (15)
C4—C5—C6—C7	−0.7 (3)	C8—C9—S1—C2	179.07 (19)
C5—C6—C7—C8	1.0 (4)	C3—C2—S1—C9	0.82 (16)
C6—C7—C8—C9	−0.5 (3)	S10—C2—S1—C9	178.86 (13)
C5—C4—C9—C8	0.5 (3)	C3—C2—S10—C11	−84.8 (2)
C3—C4—C9—C8	−179.35 (18)	S1—C2—S10—C11	97.56 (14)
C5—C4—C9—S1	−179.67 (14)	C16—C11—S10—C2	−19.9 (2)
C3—C4—C9—S1	0.5 (2)	C12—C11—S10—C2	161.70 (18)
C7—C8—C9—C4	−0.3 (3)		

### 3-Iodo-2-(phenylsulfanyl)benzo[b]thiophene (2) Crystal data

**Table:**

C_14_H_9_IS_2_	*F*(000) = 712
*M**_r_* = 368.23	*D*_x_ = 1.863 Mg m^−^^3^
Monoclinic, *P*2_1_/*c*	Mo *K*α radiation, λ = 0.71073 Å
*a* = 8.4872 (3) Å	Cell parameters from 9841 reflections
*b* = 9.9629 (4) Å	θ = 2.6–27.5°
*c* = 15.6485 (7) Å	µ = 2.73 mm^−^^1^
β = 97.052 (1)°	*T* = 294 K
*V* = 1313.18 (9) Å^3^	Parallelepiped, colourless
*Z* = 4	0.16 × 0.13 × 0.05 mm

### 3-Iodo-2-(phenylsulfanyl)benzo[b]thiophene (2) Data collection

**Table:**

Bruker D8 Venture/Photon 100 CMOS diffractometer	3001 independent reflections
Radiation source: fine-focus sealed tube	2585 reflections with *I* > 2σ(*I*)
Graphite monochromator	*R*_int_ = 0.045
Detector resolution: 10.4167 pixels mm^-1^	θ_max_ = 27.5°, θ_min_ = 2.6°
φ and ω scans	*h* = −11→11
Absorption correction: multi-scan (SADABS; Krause *et al.*, 2015)	*k* = −12→12
*T*_min_ = 0.690, *T*_max_ = 0.746	*l* = −20→20
56046 measured reflections	

### 3-Iodo-2-(phenylsulfanyl)benzo[b]thiophene (2) Refinement

**Table:**

Refinement on *F*^2^	Primary atom site location: structure-invariant direct methods
Least-squares matrix: full	Secondary atom site location: difference Fourier map
*R*[*F*^2^ > 2σ(*F*^2^)] = 0.022	Hydrogen site location: difference Fourier map
*wR*(*F*^2^) = 0.051	All H-atom parameters refined
*S* = 1.08	*w* = 1/[σ^2^(*F*_o_^2^) + (0.0218*P*)^2^ + 0.8146*P*] where *P* = (*F*_o_^2^ + 2*F*_c_^2^)/3
3001 reflections	(Δ/σ)_max_ = 0.001
190 parameters	Δρ_max_ = 0.45 e Å^−^^3^
0 restraints	Δρ_min_ = −0.85 e Å^−^^3^

### 3-Iodo-2-(phenylsulfanyl)benzo[b]thiophene (2) Special details

**Table:**

Geometry. All esds (except the esd in the dihedral angle between two l.s. planes) are estimated using the full covariance matrix. The cell esds are taken into account individually in the estimation of esds in distances, angles and torsion angles; correlations between esds in cell parameters are only used when they are defined by crystal symmetry. An approximate (isotropic) treatment of cell esds is used for estimating esds involving l.s. planes.

### 3-Iodo-2-(phenylsulfanyl)benzo[b]thiophene (2) Fractional atomic coordinates and isotropic or equivalent isotropic displacement parameters (Å^2^)

**Table:**

	*x*	*y*	*z*	*U*_iso_*/*U*_eq_	
C2	0.6667 (3)	0.8425 (2)	−0.08322 (15)	0.0350 (5)	
C3	0.7271 (2)	0.9337 (2)	−0.02410 (14)	0.0308 (4)	
C4	0.6724 (2)	1.0681 (2)	−0.04289 (13)	0.0279 (4)	
C5	0.7097 (3)	1.1876 (2)	0.00262 (15)	0.0351 (5)	
C6	0.6420 (3)	1.3057 (2)	−0.02866 (17)	0.0415 (5)	
C7	0.5388 (3)	1.3091 (2)	−0.10526 (17)	0.0434 (6)	
C8	0.5000 (3)	1.1942 (2)	−0.15099 (15)	0.0397 (5)	
C9	0.5672 (2)	1.0732 (2)	−0.11926 (13)	0.0306 (4)	
C11	0.8796 (3)	0.6562 (2)	−0.13223 (14)	0.0359 (5)	
C12	0.9622 (3)	0.5360 (2)	−0.11839 (17)	0.0459 (6)	
C13	1.1021 (3)	0.5172 (3)	−0.15346 (19)	0.0547 (7)	
C14	1.1602 (3)	0.6167 (3)	−0.20217 (19)	0.0539 (7)	
C15	1.0784 (3)	0.7353 (3)	−0.21568 (17)	0.0466 (6)	
C16	0.9382 (3)	0.7558 (2)	−0.18094 (16)	0.0408 (5)	
S1	0.53633 (8)	0.91592 (6)	−0.16492 (4)	0.04052 (14)	
S10	0.69891 (8)	0.66946 (6)	−0.08560 (5)	0.04924 (16)	
I1	0.88517 (2)	0.88685 (2)	0.08375 (2)	0.04613 (7)	
H5	0.779 (3)	1.183 (3)	0.0538 (17)	0.047 (7)*	
H6	0.667 (3)	1.387 (3)	−0.0011 (18)	0.048 (8)*	
H7	0.502 (3)	1.394 (3)	−0.1290 (19)	0.059 (9)*	
H8	0.434 (3)	1.196 (3)	−0.2021 (17)	0.049 (7)*	
H12	0.924 (3)	0.471 (3)	−0.0845 (17)	0.049 (7)*	
H13	1.162 (3)	0.435 (3)	−0.1465 (19)	0.061 (8)*	
H14	1.251 (4)	0.604 (3)	−0.229 (2)	0.071 (10)*	
H15	1.116 (3)	0.797 (3)	−0.2450 (17)	0.043 (7)*	
H16	0.882 (3)	0.829 (3)	−0.1901 (18)	0.053 (8)*	

### 3-Iodo-2-(phenylsulfanyl)benzo[b]thiophene (2) Atomic displacement parameters (Å^2^)

**Table:**

	*U*^11^	*U*^22^	*U*^33^	*U*^12^	*U*^13^	*U*^23^
C2	0.0372 (11)	0.0290 (11)	0.0406 (12)	0.0014 (9)	0.0124 (9)	0.0018 (9)
C3	0.0288 (10)	0.0313 (10)	0.0331 (11)	0.0020 (8)	0.0070 (8)	0.0060 (8)
C4	0.0265 (9)	0.0296 (10)	0.0286 (10)	−0.0001 (8)	0.0083 (8)	0.0024 (8)
C5	0.0355 (11)	0.0351 (12)	0.0347 (12)	−0.0020 (9)	0.0036 (9)	−0.0015 (9)
C6	0.0468 (13)	0.0287 (12)	0.0501 (14)	−0.0016 (10)	0.0104 (11)	−0.0024 (10)
C7	0.0490 (14)	0.0316 (12)	0.0501 (14)	0.0057 (10)	0.0089 (11)	0.0110 (10)
C8	0.0425 (12)	0.0415 (13)	0.0345 (12)	0.0017 (10)	0.0020 (10)	0.0118 (10)
C9	0.0330 (10)	0.0313 (10)	0.0281 (10)	−0.0021 (8)	0.0066 (8)	0.0019 (8)
C11	0.0447 (12)	0.0290 (10)	0.0344 (11)	−0.0015 (9)	0.0058 (9)	−0.0068 (9)
C12	0.0600 (16)	0.0310 (13)	0.0482 (15)	0.0038 (11)	0.0125 (12)	−0.0003 (11)
C13	0.0622 (17)	0.0407 (14)	0.0622 (18)	0.0121 (13)	0.0111 (14)	−0.0098 (13)
C14	0.0503 (15)	0.0624 (18)	0.0507 (16)	0.0050 (13)	0.0131 (12)	−0.0176 (14)
C15	0.0530 (15)	0.0495 (15)	0.0386 (13)	−0.0092 (12)	0.0100 (11)	−0.0004 (12)
C16	0.0479 (13)	0.0340 (13)	0.0406 (13)	0.0002 (11)	0.0052 (10)	0.0022 (10)
S1	0.0494 (3)	0.0374 (3)	0.0340 (3)	−0.0051 (2)	0.0024 (2)	−0.0054 (2)
S10	0.0561 (4)	0.0257 (3)	0.0708 (4)	−0.0024 (3)	0.0276 (3)	0.0005 (3)
I1	0.03649 (9)	0.04740 (10)	0.05267 (11)	0.00385 (7)	−0.00193 (6)	0.01649 (7)

### 3-Iodo-2-(phenylsulfanyl)benzo[b]thiophene (2) Geometric parameters (Å, º)

**Table:**

C2—C3	1.353 (3)	C8—H8	0.92 (3)
C2—S1	1.745 (2)	C9—S1	1.728 (2)
C2—S10	1.746 (2)	C11—C16	1.381 (3)
C3—C4	1.436 (3)	C11—C12	1.391 (3)
C3—I1	2.076 (2)	C11—S10	1.782 (2)
C4—C9	1.402 (3)	C12—C13	1.380 (4)
C4—C5	1.403 (3)	C12—H12	0.92 (3)
C5—C6	1.374 (3)	C13—C14	1.378 (4)
C5—H5	0.93 (3)	C13—H13	0.96 (3)
C6—C7	1.395 (4)	C14—C15	1.374 (4)
C6—H6	0.93 (3)	C14—H14	0.93 (3)
C7—C8	1.369 (4)	C15—C16	1.383 (4)
C7—H7	0.96 (3)	C15—H15	0.85 (3)
C8—C9	1.399 (3)	C16—H16	0.87 (3)
			
C3—C2—S1	111.84 (17)	C8—C9—S1	126.88 (17)
C3—C2—S10	129.12 (18)	C4—C9—S1	111.62 (15)
S1—C2—S10	119.02 (14)	C16—C11—C12	119.7 (2)
C2—C3—C4	113.64 (19)	C16—C11—S10	123.96 (19)
C2—C3—I1	123.89 (16)	C12—C11—S10	116.29 (19)
C4—C3—I1	122.46 (16)	C13—C12—C11	119.8 (3)
C9—C4—C5	118.87 (19)	C13—C12—H12	120.7 (17)
C9—C4—C3	111.34 (19)	C11—C12—H12	119.5 (17)
C5—C4—C3	129.8 (2)	C14—C13—C12	120.4 (3)
C6—C5—C4	119.2 (2)	C14—C13—H13	117.1 (18)
C6—C5—H5	122.5 (17)	C12—C13—H13	122.5 (18)
C4—C5—H5	118.3 (17)	C15—C14—C13	119.7 (3)
C5—C6—C7	121.2 (2)	C15—C14—H14	118 (2)
C5—C6—H6	121.4 (17)	C13—C14—H14	122 (2)
C7—C6—H6	117.3 (17)	C14—C15—C16	120.7 (3)
C8—C7—C6	120.9 (2)	C14—C15—H15	119.1 (18)
C8—C7—H7	119.3 (18)	C16—C15—H15	120.2 (18)
C6—C7—H7	119.5 (18)	C11—C16—C15	119.7 (2)
C7—C8—C9	118.3 (2)	C11—C16—H16	117.6 (19)
C7—C8—H8	121.6 (18)	C15—C16—H16	122.6 (19)
C9—C8—H8	120.0 (18)	C9—S1—C2	91.54 (11)
C8—C9—C4	121.5 (2)	C2—S10—C11	103.12 (11)
			
S1—C2—C3—C4	−0.9 (2)	C3—C4—C9—S1	0.7 (2)
S10—C2—C3—C4	−179.35 (17)	C16—C11—C12—C13	0.0 (4)
S1—C2—C3—I1	179.47 (11)	S10—C11—C12—C13	179.1 (2)
S10—C2—C3—I1	1.0 (3)	C11—C12—C13—C14	−0.1 (4)
C2—C3—C4—C9	0.1 (3)	C12—C13—C14—C15	0.1 (4)
I1—C3—C4—C9	179.77 (14)	C13—C14—C15—C16	0.0 (4)
C2—C3—C4—C5	−179.4 (2)	C12—C11—C16—C15	0.1 (4)
I1—C3—C4—C5	0.2 (3)	S10—C11—C16—C15	−178.93 (19)
C9—C4—C5—C6	0.2 (3)	C14—C15—C16—C11	−0.1 (4)
C3—C4—C5—C6	179.7 (2)	C8—C9—S1—C2	178.9 (2)
C4—C5—C6—C7	−0.9 (4)	C4—C9—S1—C2	−1.01 (17)
C5—C6—C7—C8	0.9 (4)	C3—C2—S1—C9	1.09 (17)
C6—C7—C8—C9	−0.3 (4)	S10—C2—S1—C9	179.73 (14)
C7—C8—C9—C4	−0.3 (3)	C3—C2—S10—C11	−83.5 (2)
C7—C8—C9—S1	179.78 (19)	S1—C2—S10—C11	98.17 (15)
C5—C4—C9—C8	0.4 (3)	C16—C11—S10—C2	−20.5 (2)
C3—C4—C9—C8	−179.2 (2)	C12—C11—S10—C2	160.48 (19)
C5—C4—C9—S1	−179.70 (16)		

### 3-Bromo-2-(phenylselanyl)benzo[b]selenophene (3) Crystal data

**Table:**

C_14_H_9_BrSe_2_	*F*(000) = 784
*M**_r_* = 415.04	*D*_x_ = 2.021 Mg m^−^^3^
Monoclinic, *P*2_1_/*c*	Mo *K*α radiation, λ = 0.71073 Å
*a* = 12.3864 (11) Å	Cell parameters from 9947 reflections
*b* = 13.6816 (11) Å	θ = 2.9–27.1°
*c* = 8.0982 (6) Å	µ = 8.33 mm^−^^1^
β = 96.398 (3)°	*T* = 297 K
*V* = 1363.82 (19) Å^3^	Parallelepiped, colourless
*Z* = 4	0.17 × 0.17 × 0.12 mm

### 3-Bromo-2-(phenylselanyl)benzo[b]selenophene (3) Data collection

**Table:**

Bruker D8 Venture/Photon 100 CMOS diffractometer	2976 independent reflections
Radiation source: fine-focus sealed tube	2208 reflections with *I* > 2σ(*I*)
Graphite monochromator	*R*_int_ = 0.060
Detector resolution: 10.4167 pixels mm^-1^	θ_max_ = 27.0°, θ_min_ = 3.0°
φ and ω scans	*h* = −15→15
Absorption correction: multi-scan (SADABS; Krause *et al.*, 2015)	*k* = −17→17
*T*_min_ = 0.543, *T*_max_ = 0.746	*l* = −10→10
43875 measured reflections	

### 3-Bromo-2-(phenylselanyl)benzo[b]selenophene (3) Refinement

**Table:**

Refinement on *F*^2^	Primary atom site location: structure-invariant direct methods
Least-squares matrix: full	Secondary atom site location: difference Fourier map
*R*[*F*^2^ > 2σ(*F*^2^)] = 0.035	Hydrogen site location: inferred from neighbouring sites
*wR*(*F*^2^) = 0.069	H-atom parameters constrained
*S* = 1.03	*w* = 1/[σ^2^(*F*_o_^2^) + (0.0201*P*)^2^ + 2.3843*P*] where *P* = (*F*_o_^2^ + 2*F*_c_^2^)/3
2976 reflections	(Δ/σ)_max_ = 0.001
154 parameters	Δρ_max_ = 0.93 e Å^−^^3^
0 restraints	Δρ_min_ = −0.90 e Å^−^^3^

### 3-Bromo-2-(phenylselanyl)benzo[b]selenophene (3) Special details

**Table:**

Geometry. All esds (except the esd in the dihedral angle between two l.s. planes) are estimated using the full covariance matrix. The cell esds are taken into account individually in the estimation of esds in distances, angles and torsion angles; correlations between esds in cell parameters are only used when they are defined by crystal symmetry. An approximate (isotropic) treatment of cell esds is used for estimating esds involving l.s. planes.

### 3-Bromo-2-(phenylselanyl)benzo[b]selenophene (3) Fractional atomic coordinates and isotropic or equivalent isotropic displacement parameters (Å^2^)

**Table:**

	*x*	*y*	*z*	*U*_iso_*/*U*_eq_	
Br1	0.27259 (4)	0.68913 (3)	−0.08792 (6)	0.06404 (14)	
C2	0.2358 (3)	0.5151 (2)	0.0834 (5)	0.0433 (8)	
C3	0.2903 (3)	0.5551 (3)	−0.0323 (5)	0.0451 (8)	
C4	0.3618 (3)	0.4929 (3)	−0.1127 (4)	0.0465 (9)	
C5	0.4294 (3)	0.5160 (4)	−0.2330 (5)	0.0632 (12)	
H5	0.431618	0.579335	−0.274038	0.076*	
C6	0.4933 (4)	0.4437 (5)	−0.2911 (5)	0.0795 (16)	
H6	0.540102	0.459228	−0.369395	0.095*	
C7	0.4887 (4)	0.3486 (5)	−0.2344 (6)	0.0819 (16)	
H7	0.531156	0.300749	−0.277171	0.098*	
C8	0.4225 (4)	0.3237 (4)	−0.1160 (6)	0.0700 (13)	
H8	0.419621	0.259777	−0.077799	0.084*	
C9	0.3605 (3)	0.3961 (3)	−0.0552 (5)	0.0496 (9)	
Se1	0.26699 (4)	0.38117 (3)	0.11070 (6)	0.05761 (13)	
Se10	0.13853 (4)	0.58065 (3)	0.21047 (6)	0.06282 (15)	
C11	0.1055 (3)	0.4715 (2)	0.3458 (4)	0.0422 (8)	
C12	0.1734 (4)	0.4475 (3)	0.4854 (5)	0.0616 (11)	
H12	0.236377	0.483315	0.515093	0.074*	
C13	0.1472 (5)	0.3698 (4)	0.5807 (6)	0.0723 (14)	
H13	0.193489	0.352550	0.674522	0.087*	
C14	0.0552 (4)	0.3181 (3)	0.5402 (6)	0.0653 (13)	
H14	0.038533	0.265737	0.606089	0.078*	
C15	−0.0132 (4)	0.3424 (3)	0.4031 (6)	0.0611 (11)	
H15	−0.076485	0.306615	0.375672	0.073*	
C16	0.0109 (3)	0.4202 (3)	0.3041 (5)	0.0475 (9)	
H16	−0.036003	0.437373	0.210910	0.057*	

### 3-Bromo-2-(phenylselanyl)benzo[b]selenophene (3) Atomic displacement parameters (Å^2^)

**Table:**

	*U*^11^	*U*^22^	*U*^33^	*U*^12^	*U*^13^	*U*^23^
Br1	0.0617 (3)	0.0504 (2)	0.0805 (3)	−0.0051 (2)	0.0103 (2)	0.0238 (2)
C2	0.043 (2)	0.0332 (17)	0.054 (2)	−0.0027 (15)	0.0087 (17)	−0.0002 (15)
C3	0.043 (2)	0.0412 (19)	0.051 (2)	−0.0057 (16)	0.0034 (17)	0.0038 (16)
C4	0.036 (2)	0.063 (2)	0.0385 (19)	−0.0047 (18)	−0.0016 (16)	−0.0023 (17)
C5	0.045 (2)	0.098 (4)	0.045 (2)	−0.002 (2)	−0.0015 (19)	0.001 (2)
C6	0.052 (3)	0.145 (5)	0.042 (2)	0.007 (3)	0.008 (2)	−0.008 (3)
C7	0.070 (3)	0.117 (5)	0.057 (3)	0.027 (3)	0.001 (3)	−0.026 (3)
C8	0.070 (3)	0.075 (3)	0.063 (3)	0.016 (2)	−0.001 (2)	−0.021 (2)
C9	0.045 (2)	0.057 (2)	0.046 (2)	0.0023 (18)	0.0009 (17)	−0.0117 (18)
Se1	0.0678 (3)	0.0342 (2)	0.0741 (3)	0.00042 (18)	0.0227 (2)	0.00167 (18)
Se10	0.0762 (3)	0.0350 (2)	0.0840 (3)	0.00590 (19)	0.0385 (3)	0.00663 (19)
C11	0.046 (2)	0.0352 (18)	0.048 (2)	0.0015 (16)	0.0148 (17)	−0.0002 (15)
C12	0.051 (3)	0.061 (3)	0.070 (3)	0.000 (2)	−0.008 (2)	−0.002 (2)
C13	0.089 (4)	0.066 (3)	0.058 (3)	0.023 (3)	−0.009 (3)	0.012 (2)
C14	0.095 (4)	0.045 (2)	0.060 (3)	0.017 (2)	0.030 (3)	0.015 (2)
C15	0.063 (3)	0.049 (2)	0.075 (3)	−0.012 (2)	0.024 (2)	−0.006 (2)
C16	0.046 (2)	0.051 (2)	0.045 (2)	−0.0014 (18)	0.0037 (17)	−0.0043 (17)

### 3-Bromo-2-(phenylselanyl)benzo[b]selenophene (3) Geometric parameters (Å, º)

**Table:**

Br1—C3	1.895 (4)	C8—H8	0.9300
C2—C3	1.333 (5)	C9—Se1	1.881 (4)
C2—Se1	1.881 (3)	Se10—C11	1.923 (3)
C2—Se10	1.894 (3)	C11—C12	1.372 (5)
C3—C4	1.435 (5)	C11—C16	1.375 (5)
C4—C5	1.391 (5)	C12—C13	1.374 (6)
C4—C9	1.404 (6)	C12—H12	0.9300
C5—C6	1.381 (7)	C13—C14	1.350 (7)
C5—H5	0.9300	C13—H13	0.9300
C6—C7	1.383 (8)	C14—C15	1.361 (6)
C6—H6	0.9300	C14—H14	0.9300
C7—C8	1.372 (7)	C15—C16	1.385 (5)
C7—H7	0.9300	C15—H15	0.9300
C8—C9	1.378 (6)	C16—H16	0.9300
			
C3—C2—Se1	111.6 (3)	C8—C9—Se1	126.1 (4)
C3—C2—Se10	126.2 (3)	C4—C9—Se1	111.7 (3)
Se1—C2—Se10	122.15 (18)	C9—Se1—C2	86.85 (16)
C2—C3—C4	117.5 (3)	C2—Se10—C11	97.51 (14)
C2—C3—Br1	120.7 (3)	C12—C11—C16	120.5 (4)
C4—C3—Br1	121.8 (3)	C12—C11—Se10	120.4 (3)
C5—C4—C9	118.4 (4)	C16—C11—Se10	119.1 (3)
C5—C4—C3	129.3 (4)	C11—C12—C13	119.2 (4)
C9—C4—C3	112.3 (3)	C11—C12—H12	120.4
C6—C5—C4	119.3 (5)	C13—C12—H12	120.4
C6—C5—H5	120.3	C14—C13—C12	121.0 (4)
C4—C5—H5	120.3	C14—C13—H13	119.5
C5—C6—C7	120.9 (5)	C12—C13—H13	119.5
C5—C6—H6	119.5	C13—C14—C15	120.1 (4)
C7—C6—H6	119.5	C13—C14—H14	119.9
C8—C7—C6	121.0 (5)	C15—C14—H14	119.9
C8—C7—H7	119.5	C14—C15—C16	120.4 (4)
C6—C7—H7	119.5	C14—C15—H15	119.8
C7—C8—C9	118.1 (5)	C16—C15—H15	119.8
C7—C8—H8	120.9	C11—C16—C15	118.9 (4)
C9—C8—H8	120.9	C11—C16—H16	120.5
C8—C9—C4	122.2 (4)	C15—C16—H16	120.5
			
Se1—C2—C3—C4	−0.7 (5)	C5—C4—C9—Se1	178.4 (3)
Se10—C2—C3—C4	178.9 (3)	C3—C4—C9—Se1	−0.9 (4)
Se1—C2—C3—Br1	179.03 (19)	C8—C9—Se1—C2	179.9 (4)
Se10—C2—C3—Br1	−1.4 (5)	C4—C9—Se1—C2	0.5 (3)
C2—C3—C4—C5	−178.2 (4)	C3—C2—Se1—C9	0.1 (3)
Br1—C3—C4—C5	2.1 (6)	Se10—C2—Se1—C9	−179.5 (2)
C2—C3—C4—C9	1.1 (5)	C3—C2—Se10—C11	−177.6 (4)
Br1—C3—C4—C9	−178.6 (3)	Se1—C2—Se10—C11	1.9 (3)
C9—C4—C5—C6	−0.4 (6)	C16—C11—C12—C13	1.7 (6)
C3—C4—C5—C6	178.8 (4)	Se10—C11—C12—C13	179.3 (3)
C4—C5—C6—C7	1.7 (7)	C11—C12—C13—C14	−1.0 (7)
C5—C6—C7—C8	−1.6 (8)	C12—C13—C14—C15	0.1 (7)
C6—C7—C8—C9	0.1 (7)	C13—C14—C15—C16	0.0 (7)
C7—C8—C9—C4	1.2 (6)	C12—C11—C16—C15	−1.5 (6)
C7—C8—C9—Se1	−178.2 (3)	Se10—C11—C16—C15	−179.2 (3)
C5—C4—C9—C8	−1.0 (6)	C14—C15—C16—C11	0.7 (6)
C3—C4—C9—C8	179.6 (4)		

### 3-Iodo-2-(phenylselanyl)benzo[b]selenophene (4) Crystal data

**Table:**

C_14_H_9_ISe_2_	*F*(000) = 856
*M**_r_* = 462.03	*D*_x_ = 2.175 Mg m^−^^3^
Monoclinic, *P*2_1_/*c*	Mo *K*α radiation, λ = 0.71073 Å
*a* = 12.9606 (6) Å	Cell parameters from 9830 reflections
*b* = 13.5999 (7) Å	θ = 3.0–27.9°
*c* = 8.0448 (4) Å	µ = 7.40 mm^−^^1^
β = 95.585 (2)°	*T* = 292 K
*V* = 1411.27 (12) Å^3^	Parallelepiped, colourless
*Z* = 4	0.51 × 0.47 × 0.24 mm

### 3-Iodo-2-(phenylselanyl)benzo[b]selenophene (4) Data collection

**Table:**

Bruker D8 Venture/Photon 100 CMOS diffractometer	3391 independent reflections
Radiation source: fine-focus sealed tube	2702 reflections with *I* > 2σ(*I*)
Graphite monochromator	*R*_int_ = 0.054
Detector resolution: 10.4167 pixels mm^-1^	θ_max_ = 28.0°, θ_min_ = 3.0°
φ and ω scans	*h* = −17→17
Absorption correction: multi-scan (SADABS; Krause *et al.*, 2015)	*k* = −17→17
*T*_min_ = 0.390, *T*_max_ = 0.746	*l* = −10→10
54241 measured reflections	

### 3-Iodo-2-(phenylselanyl)benzo[b]selenophene (4) Refinement

**Table:**

Refinement on *F*^2^	Primary atom site location: structure-invariant direct methods
Least-squares matrix: full	Secondary atom site location: difference Fourier map
*R*[*F*^2^ > 2σ(*F*^2^)] = 0.037	Hydrogen site location: difference Fourier map
*wR*(*F*^2^) = 0.083	All H-atom parameters refined
*S* = 1.04	*w* = 1/[σ^2^(*F*_o_^2^) + (0.030*P*)^2^ + 4.781*P*] where *P* = (*F*_o_^2^ + 2*F*_c_^2^)/3
3391 reflections	(Δ/σ)_max_ = 0.001
190 parameters	Δρ_max_ = 1.14 e Å^−^^3^
0 restraints	Δρ_min_ = −1.82 e Å^−^^3^

### 3-Iodo-2-(phenylselanyl)benzo[b]selenophene (4) Special details

**Table:**

Geometry. All esds (except the esd in the dihedral angle between two l.s. planes) are estimated using the full covariance matrix. The cell esds are taken into account individually in the estimation of esds in distances, angles and torsion angles; correlations between esds in cell parameters are only used when they are defined by crystal symmetry. An approximate (isotropic) treatment of cell esds is used for estimating esds involving l.s. planes.

### 3-Iodo-2-(phenylselanyl)benzo[b]selenophene (4) Fractional atomic coordinates and isotropic or equivalent isotropic displacement parameters (Å^2^)

**Table:**

	*x*	*y*	*z*	*U*_iso_*/*U*_eq_	
C2	0.7614 (3)	0.4907 (3)	0.4123 (5)	0.0348 (9)	
C3	0.7104 (3)	0.4513 (3)	0.5349 (5)	0.0353 (9)	
C4	0.6366 (3)	0.5137 (4)	0.6061 (5)	0.0395 (10)	
C5	0.5726 (4)	0.4915 (5)	0.7306 (6)	0.0511 (13)	
C6	0.5065 (4)	0.5634 (6)	0.7810 (7)	0.0655 (18)	
C7	0.5049 (5)	0.6563 (6)	0.7130 (8)	0.0693 (19)	
C8	0.5669 (5)	0.6814 (5)	0.5885 (7)	0.0587 (15)	
C9	0.6321 (4)	0.6082 (4)	0.5366 (6)	0.0447 (11)	
C11	0.8883 (3)	0.5295 (3)	0.1453 (5)	0.0354 (9)	
C12	0.9754 (4)	0.5864 (4)	0.1749 (6)	0.0407 (10)	
C13	0.9950 (4)	0.6605 (4)	0.0638 (7)	0.0512 (13)	
C14	0.9290 (5)	0.6751 (4)	−0.0757 (7)	0.0569 (15)	
C15	0.8411 (6)	0.6181 (5)	−0.1068 (8)	0.0673 (17)	
C16	0.8210 (5)	0.5442 (5)	0.0037 (8)	0.0566 (14)	
I1	0.74197 (2)	0.30752 (2)	0.61759 (4)	0.04627 (11)	
Se1	0.72215 (4)	0.62182 (4)	0.36770 (7)	0.05061 (15)	
Se10	0.86334 (4)	0.42591 (4)	0.29872 (7)	0.04875 (15)	
H5	0.575 (4)	0.430 (4)	0.777 (6)	0.033 (12)*	
H6	0.465 (5)	0.541 (5)	0.859 (8)	0.08 (2)*	
H8	0.571 (5)	0.750 (5)	0.540 (8)	0.069 (19)*	
H7	0.459 (5)	0.703 (5)	0.757 (8)	0.08 (2)*	
H12	1.022 (4)	0.576 (4)	0.263 (7)	0.052 (16)*	
H13	1.057 (5)	0.701 (5)	0.085 (8)	0.068 (18)*	
H14	0.941 (6)	0.724 (6)	−0.147 (9)	0.09 (2)*	
H15	0.793 (6)	0.631 (5)	−0.196 (9)	0.09 (2)*	
H16	0.768 (5)	0.507 (5)	−0.015 (8)	0.08 (2)*	

### 3-Iodo-2-(phenylselanyl)benzo[b]selenophene (4) Atomic displacement parameters (Å^2^)

**Table:**

	*U*^11^	*U*^22^	*U*^33^	*U*^12^	*U*^13^	*U*^23^
C2	0.035 (2)	0.028 (2)	0.041 (2)	−0.0025 (17)	0.0056 (18)	−0.0003 (17)
C3	0.033 (2)	0.036 (2)	0.037 (2)	−0.0010 (17)	0.0029 (17)	−0.0001 (18)
C4	0.034 (2)	0.050 (3)	0.034 (2)	−0.0012 (19)	−0.0009 (17)	−0.0065 (19)
C5	0.041 (3)	0.072 (4)	0.039 (3)	0.004 (2)	0.003 (2)	−0.007 (3)
C6	0.044 (3)	0.111 (6)	0.042 (3)	0.007 (3)	0.008 (2)	−0.015 (3)
C7	0.062 (4)	0.090 (5)	0.054 (3)	0.033 (3)	0.000 (3)	−0.024 (3)
C8	0.064 (3)	0.059 (4)	0.051 (3)	0.023 (3)	−0.006 (3)	−0.014 (3)
C9	0.040 (2)	0.050 (3)	0.043 (3)	0.007 (2)	0.000 (2)	−0.009 (2)
C11	0.037 (2)	0.032 (2)	0.039 (2)	0.0015 (17)	0.0112 (18)	−0.0031 (17)
C12	0.040 (2)	0.042 (3)	0.040 (2)	−0.004 (2)	0.008 (2)	−0.003 (2)
C13	0.054 (3)	0.039 (3)	0.064 (3)	−0.012 (2)	0.021 (3)	−0.004 (2)
C14	0.079 (4)	0.038 (3)	0.057 (3)	0.008 (3)	0.025 (3)	0.014 (2)
C15	0.076 (4)	0.070 (4)	0.054 (3)	0.013 (3)	−0.008 (3)	0.007 (3)
C16	0.052 (3)	0.055 (3)	0.061 (3)	−0.009 (3)	−0.006 (3)	0.001 (3)
I1	0.04859 (18)	0.04025 (18)	0.05068 (19)	−0.00477 (13)	0.00843 (13)	0.01099 (13)
Se1	0.0609 (3)	0.0314 (3)	0.0616 (3)	0.0050 (2)	0.0163 (2)	0.0066 (2)
Se10	0.0558 (3)	0.0320 (3)	0.0628 (3)	0.0051 (2)	0.0282 (2)	0.0073 (2)

### 3-Iodo-2-(phenylselanyl)benzo[b]selenophene (4) Geometric parameters (Å, º)

**Table:**

C2—C3	1.351 (6)	C8—H8	1.01 (7)
C2—Se1	1.880 (4)	C9—Se1	1.885 (5)
C2—Se10	1.895 (4)	C11—C12	1.370 (6)
C3—C4	1.438 (6)	C11—C16	1.380 (7)
C3—I1	2.093 (4)	C11—Se10	1.921 (4)
C4—C5	1.393 (7)	C12—C13	1.387 (7)
C4—C9	1.402 (7)	C12—H12	0.90 (6)
C5—C6	1.386 (8)	C13—C14	1.358 (9)
C5—H5	0.92 (5)	C13—H13	0.97 (6)
C6—C7	1.377 (10)	C14—C15	1.381 (9)
C6—H6	0.92 (7)	C14—H14	0.90 (7)
C7—C8	1.386 (10)	C15—C16	1.383 (9)
C7—H7	0.96 (7)	C15—H15	0.92 (7)
C8—C9	1.396 (7)	C16—H16	0.86 (7)
			
C3—C2—Se1	111.9 (3)	C8—C9—Se1	125.5 (5)
C3—C2—Se10	125.6 (3)	C4—C9—Se1	111.9 (3)
Se1—C2—Se10	122.5 (2)	C12—C11—C16	120.4 (5)
C2—C3—C4	116.7 (4)	C12—C11—Se10	119.3 (4)
C2—C3—I1	120.5 (3)	C16—C11—Se10	120.3 (4)
C4—C3—I1	122.8 (3)	C11—C12—C13	119.8 (5)
C5—C4—C9	118.7 (5)	C11—C12—H12	122 (4)
C5—C4—C3	128.6 (5)	C13—C12—H12	118 (4)
C9—C4—C3	112.7 (4)	C14—C13—C12	119.9 (5)
C6—C5—C4	119.1 (6)	C14—C13—H13	120 (4)
C6—C5—H5	122 (3)	C12—C13—H13	120 (4)
C4—C5—H5	119 (3)	C13—C14—C15	120.7 (5)
C7—C6—C5	121.1 (6)	C13—C14—H14	120 (5)
C7—C6—H6	127 (4)	C15—C14—H14	119 (5)
C5—C6—H6	112 (5)	C14—C15—C16	119.7 (6)
C6—C7—C8	121.8 (5)	C14—C15—H15	121 (5)
C6—C7—H7	117 (4)	C16—C15—H15	119 (5)
C8—C7—H7	122 (4)	C11—C16—C15	119.5 (5)
C7—C8—C9	116.8 (6)	C11—C16—H16	119 (5)
C7—C8—H8	124 (4)	C15—C16—H16	121 (5)
C9—C8—H8	119 (4)	C2—Se1—C9	86.8 (2)
C8—C9—C4	122.5 (5)	C2—Se10—C11	97.92 (18)
			
Se1—C2—C3—C4	0.8 (5)	C5—C4—C9—Se1	−178.7 (4)
Se10—C2—C3—C4	179.3 (3)	C3—C4—C9—Se1	1.6 (5)
Se1—C2—C3—I1	−177.8 (2)	C16—C11—C12—C13	1.4 (7)
Se10—C2—C3—I1	0.7 (6)	Se10—C11—C12—C13	179.1 (4)
C2—C3—C4—C5	178.7 (5)	C11—C12—C13—C14	−1.3 (8)
I1—C3—C4—C5	−2.7 (7)	C12—C13—C14—C15	1.2 (9)
C2—C3—C4—C9	−1.6 (6)	C13—C14—C15—C16	−1.1 (10)
I1—C3—C4—C9	177.0 (3)	C12—C11—C16—C15	−1.3 (8)
C9—C4—C5—C6	0.3 (7)	Se10—C11—C16—C15	−178.9 (5)
C3—C4—C5—C6	180.0 (5)	C14—C15—C16—C11	1.1 (10)
C4—C5—C6—C7	−1.5 (8)	C3—C2—Se1—C9	0.1 (3)
C5—C6—C7—C8	1.8 (9)	Se10—C2—Se1—C9	−178.5 (3)
C6—C7—C8—C9	−0.8 (9)	C8—C9—Se1—C2	179.7 (5)
C7—C8—C9—C4	−0.5 (8)	C4—C9—Se1—C2	−1.0 (4)
C7—C8—C9—Se1	178.8 (4)	C3—C2—Se10—C11	177.6 (4)
C5—C4—C9—C8	0.7 (7)	Se1—C2—Se10—C11	−4.0 (3)
C3—C4—C9—C8	−179.1 (5)		
